# Synthesis, Bioproduction and Bioactivity of Perillic Acid—A Review

**DOI:** 10.3390/molecules30030528

**Published:** 2025-01-24

**Authors:** Thaís de Souza Rolim, André Luiz Franco Sampaio, José Luiz Mazzei, Davyson Lima Moreira, Antonio Carlos Siani

**Affiliations:** 1 Institute of Drug Technology, Fiocruz, Sizenando Nabuco St. 100, Manguinhos, Rio de Janeiro 21041-250, RJ, Brazil; thais.rolim94@gmail.com (T.d.S.R.); andre.sampaio@fiocruz.com (A.L.F.S.); jose.mazzei@fiocruz.br (J.L.M.); 2 Rio de Janeiro Botanical Garden Research Institute, Pacheco Leão St. 915, Jardim Botânico, Rio de Janeiro 22460-030, RJ, Brazil

**Keywords:** antitumor monoterpenes, perilllic scaffold synthesis, monoterpene biotransformation, perillic acid bioactivity

## Abstract

Perillic acid (PA) is a limonene derivative in which the exocyclic methyl is oxidized to a carboxyl group. Although endowed with potential anticancer activity, PA has been much less explored regarding its biological properties than analogous compounds such as perillyl alcohol, perillaldehyde, or limonene itself. PA is usually described in mixture with alcohols and ketones produced in the oxidation of monoterpenes, with relatively few existing reports focusing on the PA molecule. This study provides a comprehensive review of PA, addressing its origin, the processes of obtaining it through organic synthesis and biotransformation, and the pharmacological tests in which it is either the lead compound or reference for in vitro efficacy in experimental models. Although feasible and generally poorly yielded, the synthesis of PA from limonene requires multiple steps and the use of unusual catalysts. The most economical process involves using (−)-β-pinene epoxide as the starting material, ending up with (−)-PA. On the other hand, some bacteria and yeasts are successful in producing, exclusively or at satisfactory purity level, PA from limonene or a few other monoterpenes, through environmentally friendly approaches. The compiled data revealed that, with few exceptions, most reports on PA bioactivity are related to its ability to interfere with the prenylation process of oncogenic proteins, an essential step for the growth and dissemination of cancer cells. The present survey reveals that there is still a vast field to disclose regarding the obtaining and scaling of PA via the fermentative route, as well as extending prospective studies on its properties and possible pharmacological applications, especially in the preclinical oncology field.

## 1. Introduction

**Figure 1:**
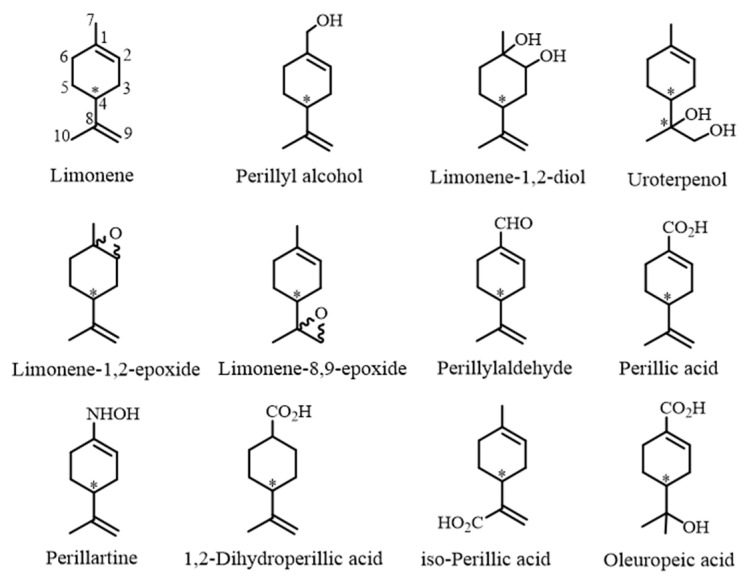
Compounds of the perillic series and relevant limonene oxidized derivatives. (*) = Carbon asymmetric center.

Perillic derivatives are monoterpenoids of the menthane group, in which the exocyclic C-7 of limonene is oxidized. The most common compounds in this series are perillyl alcohol (POH), perillaldehyde (PAL), and perillic acid (PA), in addition to perillartine (corresponding to PA oxime) (Figure 1). Regarding the perillic series, limonene, POH, and PAL have been extensively explored for their activity toward diverse pharmacological targets, as reviewed elsewhere [1,2,3]. These compounds have also been the subject of a variety of clinical studies, among which those focused on anticancer activity stand out [4]. The oxime perillartine is long known as an intense natural sweetener [5] and recently has resurfaced as subject of scientific interest for its usefulness in combating obesity-related disorders [6].

In turn, PA, which represents the highest oxidation state of the menthane’s exocyclic methyl group, is best known as one of the major metabolites that appear in the blood plasma of mammals treated with limonene, POH, or PAL [4,7]. Its ready bioavailability, evidenced from pharmacokinetic studies involving the administration of less oxidized analogs, led to the assumption that PA might either contribute to the approached bioactivities thereof [8,9] or be involved in their mechanism of action [10]. While limonene, POH, and PAL have been extensively described with respect to potential human health benefits [3,11,12], investigations focusing on PA are sparse in the literature and poorly systematized so far [13]. The present study compiles such information by approaching the PA preparation and its biological activities reported in the literature.

### 1.1. Perillic Compounds: Botanical Origin and Early Studies

The perillic compounds originate from some varieties of the species *Perilla frutescens* (L.) Britton, a Lamiaceae herb regularly cultivated for culinary purposes in Japan (*shiso*) and Korea (*kaennip*) [14], being responsible for the combined parsley–celery-like flavoring agent in oriental sauces. Also known early on based on its antifungal properties [15], the plant spread throughout South Asia [16], China [17], and India [18,19]. As it spread geographically, the species acquired morphological plasticity, as evidenced by studies involving edapho-climatic factors such as altitude and photoperiod regime [20,21]. Additionally, the volatile content of perilla plants has been defined by diverse chemotypes [22,23], according to the preferential accumulation of terpenoids other than the most frequent perillaldehyde [19,24,25].

The “perillic” nomenclature was established during the first study on the essential oil of the species at the time identified as *Perilla nankinensis* Decne (*Perilla arguta* Benth). In 1910, a sample of its raw volatile extract was sent from Yokohama to the industry of aromas Schimmel Co. in Leipzig, Germany, for chemical analysis [26]. The result of this work, published in the next year, indicated that the material contained 50% of a strongly levorotatory aldehyde (α_D_ −150), which was primarily responsible for the aromatic characteristics of the oil [27]. The suggestion by the authors to call it perillaldehyde influenced the spelling of its derivatives, given the pivotal role it played during structural elucidation. The binomial *P. nankinensis* Decne is now aggregated to *Perilla frutescens* var. crispa (Tunb.) H. Deane (wfo-0000267286; wfo-0001069837) [16,28]. Currently, the World Flora Online platform recognizes four varieties of *P. frutescens* (wfo-0000267281), into which the classifications of thirty-eight botanical synonyms are comprised.

Semmler and Zaar (1911) purified the oily aldehyde from the crude extract they received by preparing the sodium bisulfite adduct followed by alkaline release and ether extraction [27]. Its formula was determined as C_10_H_14_O. Reduction with zinc powder in acetic acid followed by acetylation led to an acetate that afforded the alcohol by hydrolysis. This alcohol was treated with phosphorus pentachloride to give a chlorinated compound that was reduced by sodium alcoholate to *l*-limonene. Furthermore, the acid C_10_H_14_O_2_ produced by air oxidation reinforced the original molecule as being an aldehyde. A second stepwise sequence involved a quantitative reaction with hydroxylamine followed by oxime reduction to nitrile with acetic anhydride and sodium acetate. Finally, the hydrolysis of the nitrile produced a compound identical to that obtained by exposing the aldehyde to air, named thereafter as perillic acid [27]. In a subsequent paper published in the same year (1911), Semmler and Zaar described the isolation of *d*-peryllaldehyde from the wood oil of the so-called “false camphor”. During the identification steps for the proposed *d*-perillaldehye, its dextrorotatory derivatives oxime, nitrile, and PA were characterized, always in comparison with the previously isolated levogyre aldehyde. In this work, the authors also claimed to have isolated myrtenal from a natural source for the first time, in addition to identifying the minor presence of *d*-limonene [29]. However, it did not result in a significant impact, since the botanical origin of the essential oil remained unknown.

Thus, both PA and perillartine were synthesized during the very first chemical approach to “Perilla extract”. However, although all of them had their formulas duly established, and alternative structures such as cuminaldehyde and myrtenal had already been properly discarded, some structural features remained to be elucidated at that time. Further investigations concerning the constitution of PAL only came to the surface by means of an erroneously translated summary from a Japanese article of 1943, published in the Chemical Abstract Service in 1947. This uncertainty was resolved by K. L. Miller during his PhD thesis, in which strong evidence for the structure of perillaldehyde as being *p*-mentha-1,8-dien-7-al was established from comparing the natural compound with the one he obtained through a multistep synthesis, starting from ethyl 4-hydroxybenzoate [30].

Perillaldehyde is not exclusively produced by *Perilla* species and may accompany the volatile limonene and POH in the essential oil composition of diverse plants [31,32,33]. The relative volatilities of these three compounds make them extractable from the plant matrices by steam distillation, hydrodistillation [34], or by less conventional techniques [35]. Occasionally, perillartine has been reported as a trace constituent in the essential oil of some *Perilla* species [36]. In turn, the non-volatile PA had its first isolation as the *O*-glycosylated form (1-β-D-glucopyranosyl-(−)-perillate, so named perilloside B), from the leaf chloroform extract of *P. frutescens*, along with two *O*-glucosides derived from *cis*- and *trans*-2,3-dihydroperillyl alcohol [37].

### 1.2. Synthesis of Perillic Acid and Analogous Compounds

Formally, PA is afforded by sequential oxidation of the limonene exocyclic methyl group (C-7, Figure 1), where POH and PAL represent intermediate states. This selective oxidation using chemical reagents is highly unfavorable through conventional chemical oxidants, considering that C-7 represents one of the five competing allylic positions in the limonene molecule. Some previous attempts in this direction are summarized below.

In general, the oxidation of limonene using traditional chromium or other metal compounds, or selenium dioxide and hydrogen peroxide, leads initially to 1,2-epoxide, which rearranges to afford complex mixtures of products, in which one or more hydroxyl groups incorporate into the menthane six-membered ring [38]. Treating limonene with organopalladium compounds in alcohol led primarily to functionalizing the isopropenyl group to linear or branched esters, depending on the catalyst used [39]. By choosing the ligands and in situ reoxidation of palladium, the functionalization can be driven to select either the exocyclic or endocyclic allylic position [40]. For instance, palladium-catalyzed oxidation of limonene, using CuCl_2_ or benzoquinone as stoichiometric oxidants, resulted in diasteromeric mixtures of carvone or carveol esters as main products [41]. Carveol ethers were also produced when limonene was oxidized by benzoquinone in alcohol and a catalytic amount of palladium (II) salt and p-toluenesulfonic acid [42]. Catalysts supported by modified silica that produce milder reactions with hydrogen peroxide, eventually susceptible to regeneration [43], or even organometallic catalysts that favor the oxidation of limonene [44] also do not solve the selectivity issue for producing perillic compounds. Nevertheless, allylstannanes were able to promote C-7 metalation in menthanes, provided that the highly competing 8,9-unsaturation was suppressed, as, for example, in 8,9-dihydrolimonene [45]. In turn, the synthesis of (+)-POH from commercial (+)-limonene has been achieved in four steps by palladium(0)-induced rearrangement of a secondary allylic acetate obtained from the (+)-1,2-limonene epoxide originated by the action of *m*-chloroperbenzoic on the starting material [46].

In fact, the earliest efforts to chemically oxidize exocyclic methyl were focused on obtaining PAL and POH, whose applications were crescent in the food and perfumery industries. In this context, obtaining PA was secondarily addressed, and most commonly in the context of the structural identification of products resulting from monoterpene oxidations. Therefore, although the subsequent oxidation of natural or synthetic POH and PAL would afford PA, this route was never systematically explored enough in the literature. A rare example includes the conversion of perillartine to methyl perillate that played a key role in the synthesis of juvabione, a compound with high juvenile hormone activity [47]. Later, PA gained attention for appearing as the main metabolite in mammalian plasma after the administration of limonene or POH [48]. To summarize, in addition to not favoring the direct preparation of perillic series through the desired oxidation of C-7, all these examples point to expensive processes, to which, in most cases, environmental costs are added [49].

A better alternative to achieve the oxidation of C-7 in the menthane nucleus came from the oxidation of pinenes. In this line, one of the first studies was published in 1950 by Ritter and Ginsburg [50], who treated α-pinene with t-butyl hypochlorite to obtain carvyl chloride and 2,6-dichlorocamphane, along with other chlorides. Alkaline hydrolysis of some of these compounds in ethanol resulted in POH through the pinane’s cyclobutane ring cleavage. This alcohol was separated from the mixture as the benzoate, which was further hydrolyzed and the free alcohol was removed from the medium by steam distillation. From this point, the investigators reproduced the pioneer experiment by Semmler and Zaar to synthesize and chemically characterize the perillaldehyde. Likewise, PA was also produced from oxime. Nevertheless, although most physical and chemical characteristics for the perillaldehyde matched the scarce data available at that time, there had not been thorough identification of the products obtained thereof.

**Figure 2:**
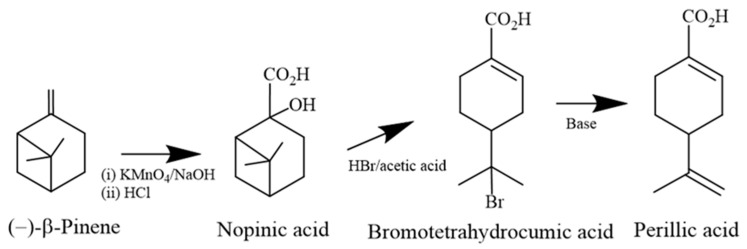
Preparation of nopinic acid and acid-catalyzed rearrangements to perillic acid [54].

Further evidence for the chemical rearrangement of pinane to a perillic framework came from the work on nopinic acid (2-hydroxy-6,6-dimethyl bicycle [3,1,1] heptane-2-carboxylic acid), a product from the oxidation of β-pinene with alkaline permanganate [51,52]. In 1962, Herz and Wahlborg refined the work performed five years earlier by P. B. Kergomard, confirming that the rearranged menthane product would depend on the type of acid employed to dehydrate the α-hydroxy acid [53]. The cleavage of the cyclobutane of nopinic acid promoted by hydrogen bromide in acetic acid, followed by the resulting halide hydrolysis with potassium hydroxide, afforded PA. Although starting from levorotatory β-pinene, the optical activity of the product was not provided [54] (Figure 2).

**Figure 3:**
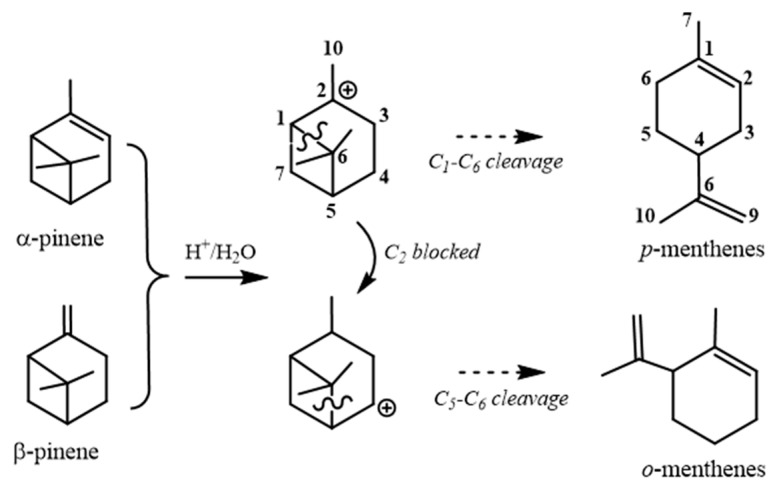
Obtaining the menthane skeleton from pinenes [55,56].

In the 1970s, the availability and low cost of turpentine contributed to consolidating the process of synthesizing menthenes from the acid-catalyzed cyclobutane ring opening in pinene derivatives. An *ortho*- or *para*-menthene framework could result from pinane derivatives, depending on the molecular features of the starting material and the reaction conditions (Figure 3) [55]. These rearrangement conditions had already been considered in previous work regarding the obtaining of fenchane, bornane, and menthane from the first carbocation generated from either α- or β-pinene, depending on the solvation conditions. In this case, the aqueous medium favored the menthane structures [56]. Such an alternative was explored to synthesize the antiparkinsonian agent (4S,5R,6R)-5,6-dihydroxy-4-(prop-1-en-2-yl)cyclohex-1-ene-1-carboxylic acid from (−)-verbenone (2-pinen-4-one) [57].

The β-pinene ring opening to menthane alcohols may be smoothly catalyzed by mercury (II) salts (as Lewis acid) in aqueous tetrahydrofuran instead of using hard mineral acids. Perillyl alcohol is afforded when employing β-pinene epoxide as the starting material [58]. Stereochemical issues regarding the rearrangement of pinane compounds under chemical catalysis or heat and light stimuli had already been previously addressed by Banthorpe and Whittaker [59].

An elegant approach was developed to selectively produce chiral liquid crystalline compounds using (S)-(−)- or (R)-(+)-perillyl alcohol, synthesized from the commercially available (S)-(−)-β-pinene and (R)-(+)-α-pinene, as building blocks. The former was epoxidized with 1 equivalent of 30% hydrogen peroxide and 0.5 equivalent of benzonitrile to give (S)-(−)-β-pinene epoxide, followed by epoxide ring opening with ammonium nitrate to afford (S)-(−)-perillyl alcohol (68% yield, 88% enantiomeric excess). With (+)-β-pinene being commercially unavailable, it was prepared by treating (+)-α-pinene with potassium *t*-butoxide and butyllithium in hexane, followed by trimethoxyborane in ethyl ether and, finally, mild hydrolysis with diluted hydrochloric acid. Both enantiomers were sequentially oxidized with manganese oxide in hexane followed by silver oxide in alkaline aqueous medium to afford PAL and PA (75–77% overall yield), without altering the C-4 chiral center [60]. The structural rearrangement of pinane-type to menthane derivatives has become a valuable strategy in the synthesis of pharmacologically active compounds. Acid-catalyzed transformations of pinane terpenoids and, more specifically, β-pinene oxide have been reviewed elsewhere [61]. More recently, a series of catalytic strategies have been proposed to trigger the β-pinene oxide four-membered ring cleavage to result in perillic derivatives [62,63,64,65,66].

Finally, it is worth mentioning that, from the 1970s onwards, many patents aiming at obtaining perillic derivatives were filled by researchers and chemical companies in several countries. Disregarding perillartine as the primary target, very few of them describe the synthesis of PA and esters as either the main target [67], side products, or even intermediates for more complex molecules [68]. Most of the claims are concerned with obtaining POH or an ester derived from it. Pinenes as a starting material are largely predominant, although nopinic acid and other menthane alcohols were eventually employed. Useful summaries of this overview can be found in Chastain et al. (1998) [69] and, specifically for POH, in Kolomeyer and Feroni (2010) [70].

### 1.3. Biotransformation of Monoterpenes to Perillic Acid and Analogous Compounds

Perillic derivatives were more straightforwardly and selectively obtained through the microbial transformation of monoterpenes, which proved quite advantageous compared to the synthetic chemistry approaches. This strategy has been used to mildly produce many molecules whose syntheses are unfavorable, laborious, and expensive. The biotransformation of terpenes is highly promising for industrial processes, given the multitude of bacteria, fungi, and yeasts capable of promoting it [71,72], delimiting a broad scope that also considers isolated enzymatic systems (eventually mutagenized) from these sources. Oxidative pathways leading to regioselective products are characteristic of cellular systems whose metabolic gear can selectively transform hydrocarbons into epoxides, alcohols and diols, aldehydes, ketones, and acids [73].

The early publications on the microbial transformation of monoterpenes were related to the biodegradation of these compounds, within the argument to investigate the redistribution of carbon in nature. In this context, the degradation of (+)-camphor by *Pseudomonas* species (isolated from sewage sludge) was addressed in the late 1950s [74]. Camphor, as the carbon source, constituted the primary model for studying the mechanisms of monoterpene biooxidation and paved the way for subsequent investigations that were published by the same group in the 1960s [75,76]. Inspired by the first of these publications and hypothesizing about the variation in the production and composition of essential oils by the action of microorganisms, the Indian group led by P. K. Bhattacharyya studied the hydroxylation of α-pinene by mold [77]. Further investigations led to the production of hydroxylated derivatives of menthane from the fermentation of a soil pseudomonad in the presence of limonene. Acidic partition of the broth led to the isolation of PA, along with a mixture of ketones, diols, and open-chain mono- and dicarboxylic acids [78,79,80,81]. The isolated compound was identified by comparing its spectroscopic data and mixed melting point with PA synthesized from nopinic acid by the method reported four years earlier by Herz and Wahlborg [54]. From this first discovery, perillic acid was increasingly reported from the fermentation of cultured microorganisms or purified enzymes in the presence of distinct monoterpene hydrocarbons, but usually occurring as side product [73], e.g., the α-terpineol metabolized by *Pseudomonas incognita* [82]. The exclusive production (or highly favored mixture with other products) of PA has been reported only in a few cases of biotransformation involving monoterpenes, mainly those using bacterial strains of *Pseudomonas putida* [83], or the yeast *Yarrowia lipolytica* [84,85]. Table 1 (in ascending order of year of publication) compiles the publications reporting the production of PA by the bioconversion of monoterpenes.

**Figure 4:**
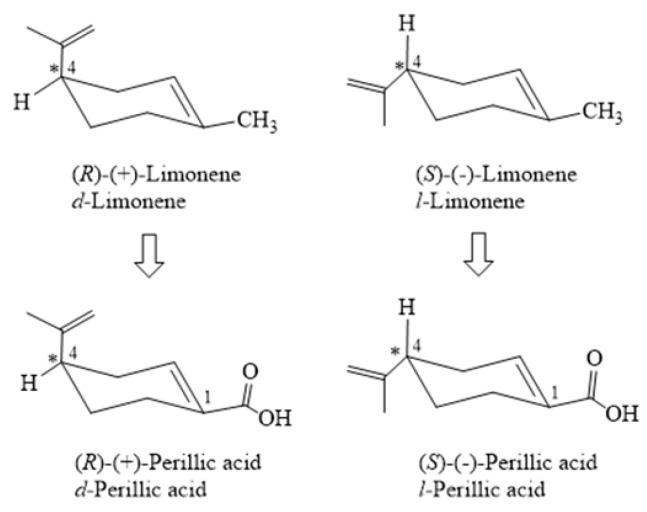
Absolute stereochemistry of limonene and perillic acid. C4 = asymmetric center [73,93,104].

Considering the chirality of the limonene molecule, it would be expected that oxidation of the exocyclic methyl by biological agents (microorganisms or enzymes) would keep the C-4 configuration of the menthane ring unaltered. It is pertinent to mention that, for limonene, studies using Raman spectroscopy demonstrated that the absolute (R) or (S) configuration of C-4 corresponded to the dextrorotatory [*d* or (+)] and levorotatory [*l* or (−)] enantiomers, respectively [104]. It can be hypothesized that the resulting PA would exhibit optical rotation in a direction like that produced by the starting material (Figure 4).

The specificity of *Pseudomonas putida* on (+)-limonene and (−)-limonene, using glycerol as cosubstrate, exclusively produced (+)-PA and (−)-PA, respectively. Chiral column gas chromatography indicated a slight optical contamination in the second case, which was attributed to impurity of the starting material [93]. Indeed, this result corroborated some earlier ones by Cadwallader et al. (1989) [88] and Miyazawa et al. (1998) [92]. The first author reported [α]^27^_D_ +138 for the specific rotation of (+)-PA produced from (+)-limonene by *Pseudomonas gladioli,* a value that was not far from the +89 found by the pioneers Dhavalikar and Bhattacharyya. The second one assumed that (+)- and (−)-limonene were biotransformed to (+)-PA and (−)-PA by *Spodoptera litura* (common cutworm) larvae intestinal bacteria. However, despite the application of accurate chromatographic and spectroscopic techniques to identify the products of limonene biooxidation, no clues about optical determination were presented. Both the levorotatory and dextrorotatory limonene were submitted to a series of bacteria from different sources, and the conversion products were analyzed by HPLC-MS. Specifically, in cases where perillic acid was found in the resulting mixtures, differences were observed in the bioconversion effectiveness. Depending on the strain, the relative rates to convert *l*- and *d*-limonene were variable. As an example, a mutant strain of *Rhodococcus erythropolis* converted *l*-limonene to *l*-PA twice as efficiently compared to the d-form. In turn, *Mycobacterium* sp. converted only *d*-limonene in low yield [95]. Following these findings, the subsequent studies employing (+)-limonene were assumed to exclusively produce (+)-PA, without any concern about a thorough confirmation of its specific optical rotation [94,96]. This inference is extendible to PA obtained from fermentation of (+)-limonene by the yeast *Yarrowia lipolytica* [84,85,103]. The literature covering the biotransformation of limonene is highly centered around the dextrorotatory form [83], given its abundant availability worldwide as side product from industries based on citrus species [105].

### 1.4. Biological Properties of Perillic Acid

Scientific attention turned to perillic derivatives after the discovery of their ability to inhibit enzymes that promote the prenylation of oncogenic proteins, thus blocking their association with cell membranes and other proteins [106,107]. Protein prenylation is a lipid post-translational modification that occurs in eukaryotes to make nascent hydrophilic proteins fully functional [108,109]. It is characterized by the addition of an isoprenoid moiety to cysteine residues near the carboxyl terminal of numerous proteins [110]. For instance, this modification brings to the protein the necessary affinity for the lipid bilayer [109], promoting efficient anchoring on plasma membranes or organellar membranes [108]. The hydrophobic moiety makes them capable of recognizing and interacting with other proteins and, when anchored in the membrane, they are responsible for playing critical roles in cell signaling [111]. The prenylation process is catalyzed by prenyltransferases (PTases) which, in humans, are mainly represented by types of farnesyltransferase (FTase) and geranylgeranyltransferase (GGTase) [112]. They transfer, respectively, farnesyl (15-carbon) and geranylgeranyl (20-carbon) groups to protein via a stepwise process [108].

The finding that prenylation is required for the activity of many oncogenic proteins [113], including some members of the Ras family, has brought a new perspective to the fight against cancer [114]. Ras (rat sarcoma virus) proteins are low-molecular-weight proteins that are expressed in all animal cell lineages and organs [115]. They are involved in cell growth, survival, and differentiation. Synthesized in inactive form in the cytosol, all Ras proteins possess in their structure an invariant cysteine residue, to which a farnesyl isoprenoid will be covalently attached under FTase mediation, to facilitate membrane association. Since the Ras protein is a common target of mutations associated with several types of cancer, the inhibition of FTase (and GGTase) has been established as a pharmacological target to be explored for anticancer drug discovery [116]. Once capable of interfering with these proteins, some terpenoids have been investigated in several molecular oncogenic processes, becoming valuable compounds applied to drug discovery [117].

Studies in this area have not only led to the exploration of monoterpenes as blockers of these key enzymes but also contributed to establishing some of these substances as the basis for preclinical bioassay models. Advanced examples exploring this target include recent clinical studies involving perillyl alcohol administered by inhalation for treatments of glioblastoma [4,118]. PA (along with dihydroperillic acid) promptly appears as the major circulating metabolite in human or rodent plasma after administering POH [9] or limonene [48,119]. The rapid metabolization of the former led to the hypothesis that PA, as the main metabolite, could be the actual active compound [8]. Such an assumption boosted the in vitro investigation on PA’s cytotoxic potential.

The compiled bioactivities of PA are displayed in Table 2, which was built in ascending order of year of publication and maintained the target (first column) as cited in the original reference. This information available for PA is much scantier than for the less oxidated limonene and POH.

## 2. Conclusions and Perspectives

The present survey corroborated the lower amount and inconspicuous information reported for PA compared to limonene, perillyl alcohol, and perillaldehye, since it is often described in complex mixtures with other oxidated monoterpenes resulting from its preparation. Nevertheless, the compilation presented herein indicates that the PA synthesis from β-pinene, which is abundant in many natural essential oils, is a valuable option for industrial exploitation. Oxidation of perillaldehyde separated from plants would be an alternative, provided that a cost–benefit relationship is met. These two routes would result in the production of levorotatory PA. On the other hand, the more suitable process reported hitherto for producing dextrorotatory PA is the biotransformation of (+)-limonene, or orange oil, by selective microorganisms, including *Pseudomonas* spp. and the yeast *Yarrowia lipolytica*. The latter has the advantage of being a biological resource that is well suited to the industrial bioproduction of foods and pharmaceuticals. In this sense, the experiment using a 2 L bioreactor and limonene fed-batch culture described by Knopp et al. may be a suitable starting point.

The array of bioactivities described for PA is largely represented by the ability to block enzymes that catalyze protein prenylation. This activity has supported trials related to the investigation of its properties as an anticancer compound. Thus, cytotoxic, antiproliferative, antimetastatic, and apoptotic properties predominate in the reports compiled for PA (72%), of which 35% address the inhibition of protein prenylation. Immunomodulatory, antiviral, and antidiabetic activities were relevant to completing the panel. Overall, the data in Table 2 indicate that PA is less potent in inhibiting protein prenylation when compared to other compounds, such as perillyl alcohol or limonene, both of which have already been the subject of clinical studies. In this sense, the low relative progress in the preclinical pharmacology of AP could be related to its lower efficacy. Further study is required to deepen the positive results achieved so far from an oncological perspective. Furthermore, it is necessary to expand biological targets to determine other useful applications for PA. For example, its eventual antibacterial and antifungal properties have so far been ignored, in view of the wide range of active menthane-type monoterpenes that even include limonene, POH, and PAL. The antifungal activity of PA, suggested 100 years ago, paradoxically represents a new field to be explored. In all these contexts, PA presents a comparative advantage over other derivatives of the perillic series because it is more soluble in aqueous media. This bonus can still be further enhanced by the possibility of PA easily providing ionic species.

## Figures and Tables

**Table 1:** Biotransformation of monoterpenes to perillic acid by bacterium (B), enzyme (E), fungus (F), yeast (Y), or insect (I).

Microorganism (B, F, I, Y)	Substrate	Bioconversion to Perillic Acid	Reference
Soil Pseudomonad (B)	limonene	Culture optimized in agar slants. Cultured plateau with 0.6 mL limonene/100 mL medium, 24 h. Incubation: rotary shaker, 3 days, 30 °C, stepwise addition of limonene up to 72 h. Acidic extract (pH 2.5) methylated, partitioned with solvents and chromatographed in alumina. PA isolated by saponification.	[78]
Pseudomonas sp. (B)	α-pinene	Like in the above line. α-Pinene (0.3–0.5%) replenished at every 24 h. Broth extractions with solvents of increasing polarity (neutral/acidic). Acidic fraction: successive column chromatography. PA in complex mixture of products. Identification by running vapor-phase chromatography with authentic sample.	[81]
Undescribed	limonene	Study of the enzymatic pathways involved in the metabolism of limonene in cells cultured with glucose: allylic oxygenation; oxygenation at the 1,2-double bond; oxidation of the C-7 methyl to PA. Further hydration, dehydrogenation, and hydrolysis lead to more oxidized PA.	[79]
Soil Pseudomonad (B)	limonene	On pinene-adapted cells, 0.3% to 0.6% *v*/*v* limonene in shake flask, 28 °C, 72 h (24 h and 48 h replenishment). Six extracts prepared from broth liquor by different solvents under neutral x acidic condition. Complex mixtures obtained. PA separated from methanolic partition of n-butanol extract and characterized as methyl ester. Saponification and comparison with authentic sample.	[80]
Soil Pseudomonad/purified aldehyde dehydrogenase (B, E)	limonene	Progressive oxidation of the C7 methyl group to the carboxyl (POH > PAH > PA). Yield or selectivity not confirmed.	[86]
Pseudomonas maltophilia (S201-1, soil) (B)	α-pinene	PA was isolated from the acidic fraction of the culture broth by column chromatography along with many other p-menthane alcohols. PA showed to be identical with sample prepared by oxidation of perillaldehyde with silver oxide (Zaar, 1911) [27]. Yield not confirmed.	[87]
Pseudomonas incognita (PI)/cell-free extract (CFE) (B)	α-terpineol (T) limonene (L) perillyl alcohol (POH)	Qualitative study on microorganism growth and metabolites produced by CFE (enzymes). PI converted T to 1-hydroxy-PA. CFE from T-adapted cells + NADH: (i) L converted to PA; (ii) + NAD+-specific dehydrogenases: POH converted to PA. Separated analysis of neutral x acidic (methyl esters) metabolites. NMR monitoring.	[82]
Pseudomonas gladioli (B)	(+)-limonene	Fermentation (4–10 days in shake flasks at 25 °C; pH 6.5; 1.0% limonene). Broth ethereal extract > neutral and acidic partition. (+)-PA identified along with (+)-α-terpineol. Only the latter resisted further biodegradation in the medium. On the 4th day: maximum of 1861 ppm for PA by quantitative HPLC.	[88]
Pseudomonas strain PL (B)	α-pinene	Literature approach on putative enzymes that open the cyclobutene ring to afford menthane scaffold, followed by oxidation of the C7 methyl group (C10 of α-pinene), ultimately leading to PA.	[89]
E. coli XL-1 cloned from Bacillus stearothermophilus BR388 (B)	perillyl alcohol	Cloned Escherichia coli transformant EC409A afforded PA (230 mg/mL) plus perillaldehyde (36 mg/mL).	[90]
Rhodococcus ruber DSM 8316 or mutants; Micrococcus roseus	limonene	Claim for production of alcohol, aldehyde, or acid from vinylic alkyl compounds by biotransformation based on screening of various microorganisms. PA (case) yield not provided.	[91]
Spodoptera litura (cutworm fed 1 mg/g of diet) (I)	(+)-limonene/ (−)-limonene	(+)-PA (43%) and (−)-PA (44%) produced along with (+)- and (−)-uroterpenol, accordingly, indicating little difference in metabolic pathway between the (+) and (−) forms. Frass extraction with CH2Cl2; neutral and acidic partition; GC-MS identification and quantitation by relative peak area.	[92]
Pseudomonas putida (solvent-resistant strain) (B)	(+)-limonene/ (−)-limonene	Emulsified limonene (150 mM) and glycerol as cosubstrate (50 mM) plus ammonia or urea as nitrogen source (pH 7.0; 30–34 °C) produced up to 18 mM (3.0 g/L) of PA. Optical properties maintained in bioconversion products. Limonene > 500 mM did not enhance PA amount.	[93]
Pseudomonas putida GS1 (B)	limonene	Fed-batch culture with non-limiting amounts of glycerol, ammonium, and limonene. PA yields (up to 64 mM; 11 g/L) were higher than other P. putida modified strain. PA extracted with ether after alkaline partition.	[94]
From screening: 10 effective strains including Rhodococcus sp. and Mycobacterium sp. (B)	d-limonene or l-limonene	Bacterial cells may be recombinant and mutagenized. Microbial cells or lysate. Collateral PA formed during POH regiospecific production. Optical activity maintained in the alcohol. Best results for PA: R. erythropolis (12 µM) and Mycobacterium sp. (190 µM). Rodococcus sp. ALK2-C7 produced only PA (specific enzymatic activity 0.3 U g/dry wt).	[95]
Pseudomonas putida DSM 12264 (B)	R-(+)-limonene (≥96%)	Integrated bioprocess that overcame product inhibition: in situ product recovery based on anion exchange Amberlite IRA 410 CI, coupled to the bioreactor and product removal strategy, led to cumulative PA of 187 mM (31 g/L) after 7 days.	[96]
Pseudomonas putida KT2440 (P)	(S)-limonene	P1: model for whole-cell-based CYP153A6 catalysis. PA and PAL constituted up to 26% of total oxidized terpenes. Study focused on POH production.	[97]
Yarrowia lipolytica ATCC 18942 (Y)	R-(+)-limonene	PA (564 mg/L) was the sole product obtained (buffered pH 7.1; 25 °C; 48 h) by stepwise addition of limonene. Glucose or surfactant addition to the medium did not improve the process.	[84]
Penicillium nigricans (F)	Δ3-carene	Byrde medium (pH 7.0). Substrate (0.6%) added stepwise (96 h). Inoculum incubation (120 h, room temperature). Extraction with ethyl acetate; residue partitioned in acidic and neutral fractions followed by chromatography. PA and other acidic compounds identified by GC-MS after methylation.	[98]
Yarrowia lipolytica (Y)	R-(+)-limonene/orange essential oil	For PA production: bioconversion parameters optimized by (i) fractional factorial design and (ii) central composite design. Accumulation of 0.368 g∙L−1 of PA (molar yield 23.1%) from 0.16% (*v*/*v*) limonene at 24 h. Subsequent substrate addition doubled PA concentration (0.793 g/L, 24.2%). Use of orange essential oil increased both PA accumulation and yield (0.872 g/L, 29.7%).	[85]
Yarrowia lipolytica (Y)	R-(+)-limonene/orange oil	PA yield: from orange oil (89.1% limonene) = 866 ppm; from commercial limonene (97% purity) = 862 to 897 ppm. No other product formed.	[99]
Pseudomonas putida GS1, recombinant Pseudomonas taiwanensis VLB120 (B)	R-(+)-limonene	In situ PA removal promoted by oxygen limitation relief and membrane-mediated substrate supply. Wild-type P. putida GS1 encoding the enzymes for limonene bioconversion, supplied with glycerol, reached 34 g/Ltube/day. Recombinant P. taiwanensis VLB120 (harboring p-cymene monooxygenase and p-cumic alcohol dehydrogenase) was 10-fold lower.	[100]
Yarrowia lipolytica (Y)	R-(+)-limonene	Fermentation medium: 40 g/L cane molasses; 10 g/L peptone; 1000 mL water; pH 4.0–6.0; 48 h. Production of up to 407 mg/L of PA.	[101]
Biocatalyst (dehydrogenases F-ALDH, ALD-S1 and ALD-S2) (E)	perillaldehyde	Tests under pre-optimized conditions for efficiency and selectivity: PAL (1 mM) + NAD+ (1 mM), biocatalyst vortexed in buffer. Incubation (24 h, 25 °C). HPLC monitoring. Biocatalysts ALD-S1 and ALD-S2 converted 80% of the substrate to PA.	[102]
Yarrowia lipolytica (Y)	R-(+)-limonene/orange essential oil	Optimized conditions for limonene-rich essential oil [(g/L): 22.9 glucose; 7.7 peptone; 4.1 yeast extract and 1.0 malt extract, resulted in 13.0 g/L and 0.18 g cell/L/h] for PA. Cell mass enhanced to 18.0 g/L in 2 L-bioreactor. PA yields: 840 mg/L from limonene and 806.4 mg/L from orange oil.	[103]

**Table 2:** Bioactivities described for perillic acid.

Activity/Model	Experimental	Outcomes	Reference
Inhibition of isoprenylation of oncogenic proteins/in vitro	NIH3T3 and M600B cells extracts labeled with [2-^14^C]mevalonolactone and test sample (0–5 mM) subjected to SDS-PAGE; isoprenylated proteins visualized by fluorography (5 days, bands 22–26 kDa)	PA (and DHPA) selectively inhibits protein isoprenilation (p21-ras and others). Methyl esters inactive at 1 mM.	[120]
Proliferation inhibition/in vitro	Inhibition of NIH3T3 cell growth by PA (1 to 5 mM).	Concentration-dependent inhibition from 15% (500 µM) to 100% (3 mM), with IC_50_ 1.3 mM.	[121]
Insulin secretion modulation/ex vivo	Pancreatic islets isolated from male Sprague Dawley rats were chemically modulated (4 substances tested) for 18 h and during subsequent secretion stages. PA (0.5–5.0 mM) tested as insulin secretion inhibitor based on its ability to block isoprenylation of small GTP-binding proteins (GBPs).	PA inhibits in a concentration-dependent manner the induction of insulin secretion by glucose at 16.7 mM (<90%) or by oxo-4-methylpentanoic acid at 15 mM (<60%). Effect dissipated when PA is only present in the culture medium. Complementary tests indicated blocking of isoprenylation of small GBPs.	[122]
Inhibition of oncoproteins farnesylation/in vitro	PA (x lovastatin): Effects on the growth of Ha-ras (WB-ras) and ras-transformed (R3611-3) and non-transformed (WB-neo and RLEC-2) viruses. Tests (0.25–2.5 mM) in rat liver epithelial cells, determined by clonal assay.	PA inhibited WB-ras cells, RLEC-2 (up to 80%), and R3611-3 (up to 50%) cell growth, but not WB-neo cells at the tested concentrations. It does not involve Ras alteration in the plasma membrane. Lovastatin reduced the growth of WB-ras by a different mechanism, increasing the cytosolic levels of Ras.	[123]
Protein isoprenylation inhibition in lymphocytes/in vitro	PA (0.5–5 mM): effects on PBMCs (stimulated or not with phytohemagglutinin) to demonstrate the importance of isoprenylation for DNA synthesis and cell cycle progression. PA (2 mM) tested in the presence and absence of mevalonate or compactine. Parallel tests performed with other non-steroidal isoprenoids. Flow cytometry analysis.	PA (2.5 mM) selectively inhibits isoprenylation of 21–26 kDa proteins. Suppression of [3H]-mevalonate incorporation into proteins results in dose-dependent inhibition (up to 75%) of DNA synthesis, stopping cell cycle in G1 and preventing entry into the S phase. Stimulated lymphocytes treated with PA: G0/G1 = 80–91%. Mevalonate does not restore lymphocyte proliferation blocked by PA.	[124]
Inhibition of intracelular proteins farnesylation/in vitro	Testing lovatastin: PBMCs cultured with PA for p21 ras inhibition, to support the theory that lovastatin affects IL-6 mRNA expression, interleukin-6 (IL-6) and leukotriene B4 synthesis.	PA caused a concentration-dependent inhibition of phytohemagglutinin-stimulated PBMC proliferation in mevalonate-loaded PBMCs treated with lovastatin.	[125]
Enzimatic inhibition/in vitro	Farnesyltransferase (PFT) and geranylgeranyltransferase (PGGT) purified from bovine brain and from *S. cerevisiae* tritium-labeled PFT. PA tested up to 10 mM for inhibitory activity.	PA: weak inhibitor (10%) of PFT and PGGT (IC_50_ > 1 mM), in contrast to the high potency of its methyl ester.	[126]
Proliferation inhibition/in vitro	Inhibition of PANC-1 (human pancreatic carcinoma) cell growth.	S-(−)-AP methyl ester (1 mM) induces 25% cell inhibition of cell growth.	[127]
Apoptosis induction/in vitro	Proximal tubular cells isolated from C57BL6 mouse kidneys. Incubation (24 h) with PA in the presence or absence of isoprenylation inhibitor. Evaluation of DNA fragmentation by nucleic acid electrophoresis.	PA increases DNA fragmentation at 5 mM, indicating inhibition of FTPase and GPTase. Geranylgeranylation is a critical step for apoptosis induction.	[128]
Apoptosis induction/in vitro	PBMCs and T cells stimulated with 2 mg/mL of mitogen in the presence of PA (0–2500 µM, 4 to 72 h). Cytokine levels determined by enzyme-linked immunosorbent assay.	PA significantly suppresses IL-2 levels during treatment and strongly reduces (90%) IL-10 production. IL-6 and latent TGF-β1 are not affected. PA disrupts signaling through the Ras/MAPK pathway, depletes farnesylated Ras, and activates T cells.	[129]
Proliferation inhibition/in vitro	Breast carcinoma T-47D, MCF-7 and MDA-MB-231 cell lines were treated with PA for 3 to 7 days before DNA assessment by fluorometric assay (3,5-diaminobenzoic acid·2HCl).	Concentration-dependent growth inhibition of T-47D 17 (90% at 10 µM to 3 mM), MCF-7 (16 to 66% at 50 µM to 3 mM) and MDA-MB-231 (26%, 3 mM). Inhibition was associated with a decrease in cells in the S phase and accumulation of cells in the G1 phase, preceded by a reduction in cyclin D1 mRNA levels.	[130]
Inhibition of Ras-prenilation/in vitro	Bullfrog sympathetic B neurons treated with PA (0.1–1.0 mM) for 6 days in the presence or absence of 200 ng/mL nerve growth factor (NGF, positive control) with mensuration of Ba^2+^ current density (IBa).	PA attenuated the effect of NGF on IBa in a concentration-dependent manner (58% to 63%). Attenuation of the NGF effect by PA and α-hydroxyfarnesylphosphonic acid. Distinct biochemical mechanisms suggest a farnesylation-dependent transduction. Evidence of Ras/MAPK involvement in Ca^2+^ channel regulation.	[131]
Apoptosis, atherosclerosis/in vitro	Inhibition of HMG-CoA-induced apoptosis in rat vascular smooth muscle cells (VSMCs) by atorvastatin in the presence of survival factor. Role of protein prenylation was assessed by exposing VSMCs to PA (2–10 mmol/L). Atherosclerotic lesions assessed by morphological criteria, annexin V binding, and DNA fragmentation. Hypodiploid cell quantification by flow cytometry.	Apoptosis contributes to preventing neointimal arterial thickening. It is induced by atorvastatin in dose-dependent manner. In the presence of atorvastatin + mevalonate, PA weakly but significantly and dose-dependently inhibits farnesylation and geranylgeranylation of low-molecular-weight proteins in VSMCs.	[132]
Inhibition of protein isoprenylation/in vitro	Inhibition of FTase and GGTase I in the rat brain cytosol: (R)-PA and (S)-PA were tested, based on the metabolites detected in human plasma after limonene ingestion.	IC_50_ (mM): 8.1 (*R*-PA), 10.7 (*S*-PA) (FTase I); 3.4 (*R*-PA), 4.1 (*S*-PA) (GGTase I). A new active metabolite described: iso-PA.	[133]
Induction of apoptosis and metabolites analysis/in vitro, in vivo	PA, POH, and PAL tested. Apoptosis of rat pheochromocytoma cells (PC12) was determined by cell cycle analysis, cellular staining, and flow cytometry. S9 (microsomes and cytosol) extracted from rat liver for enzymatic assays.	PA did not demonstrate apoptotic effects. Perilaldehyde (>200 mM) and perillyl alcohol (>500 mM) were active in high concentrations.	[134]
Proliferation inhibition/in vitro	Effect of (S)-PA (1 to 3.5 mM) on the proliferation of diploid smooth muscle cells (SMCs) from rat aorta as strategy to investigate mechanisms related to PFTase and PGGTases I and II proteins. Cell counting and DNA synthesis.	S(−)-PA (2.5 mM) reduces SMC proliferation by 65% in a concentration-dependent manner. It alters protein prenylation and blocks cell cycle progression at the G0/G1 phase and inhibits up to 70% of farnesol and geranylgeraniol incorporation into cellular proteins. Does not involve apoptosis (morphological criteria).	[135]
Proliferation inhibition/in vitro	Effect of PA (0–2.5 mM) on HTC-116 cancer cells (human colon) with analysis of the cell cycle. In situ cellular DNA measurements, Western blot and RT-PCR.	Dose-dependent inhibition (up to 90%) of cell growth correlated with G1-phase cycle blockade, via (i) increased expression of the cdk inhibitor p21Waf1/Cip1 and cyclin E and (ii) negative regulation of cyclin-dependent kinase.	[136]
Apoptosis/in vitro	PA (1 to 4 mM, 72 h) tested as apoptosis inducer in U266 cells (human multiple myeloma), with enhanced Fas ligand expression and RPMI 8226/S (human peripheral blood B lymphocytes), in parallel with a known apoptosis inducer that inhibits FTase.	PA, an FTase inhibitor, induces apoptosis independent of caspase-8/death receptor signals, as there was no significant increase in caspase-8 activity. Viable cells were reduced by 55–35%.	[137]
Immunomodulation/in vivo	Balb/c mouse treated i.p. with PA (5 × 50 µmoles/Kg bw). Analysis performed at 24 h. Parameters: (i) bone marrow cell count, (ii) circulating antibody titre, (iii) plaque-forming cells in the spleen, (iv) delayed-type hypersensitivity (DTH) induced by antigen.	(i) At 14,437.5 cells/mm^3^, peak observed on the 9th day, with no significant change in differential count, body weight, or hemoglobin content; (ii) bone marrow cells: 25.6 × 10⁶/femur, with a significant increase in cells positive for α-esterase activity (1255.3/4000 cells); (iii) increase in antibodies (512/4000 on the 12th day); (iv) 596/10⁶ pancreatic cells after the 5th day of immunization; (v) low DTH reaction with slight increase in paw thickness (0.23 to 0.25 cm).	[138]
Metastasis inhibition/in vivo	Treatment with PA (10 × 50 µmoles/Kg body weight, ip): lung metastasis in C57BL/6 mice induced by B16F10 melanoma cells. Tumor nodule measurements, complemented with uronic acid, sialic acid levels in serum and histopathological studies.	PA reduced nodule formation by 67%. Sialic acid decreased by 58% compared to control (from 126.8 to 53.6 µg/mL). Uronic acid levels were inhibited by 39.7%. Histopathology confirmed these results.	[139]
Cytotoxicity/in vitro	Pre-treatment (72 h) with PA (1.0 mM) followed by exposure to a radiation dose (1–2 Gy). Cells used: HTB-43 cancer cells (larynx), SCC-25 cells (squamous cell carcinoma) and BroTo cells (carcinoma). Calculation of Apoptotic Index.	POH or PA alone minimally affected cell viability and proliferation. Inhibition after irradiation (1 and 2 Gy): HTB-43 (50% or 71%), SCC-25 (55% or 68%), and BroTo (18% or 53%). Susceptibility: HTB-43 ≥ SCC-25 > BroTo. The reduction in viability is due to apoptosis.	[140]
Immunomodulation and proliferation inhibition/in vitro, in vivo	(i) PA (0–2500 µM, 6 doses) on NO production by peritoneal macrophages (from naive or lymphoma-inoculated Balb/c mice) stimulated with recombinant murine IFN-γ and 2.5 µg/mL of LPS for 24 h. (ii) PA (0–25 µM, 5 doses) on proliferation of splenic T lymphocytes (normal vs. lymphoma-bearing mice) induced by Con-A (flow cytometry). In vivo: delayed-type hypersensitivity reaction to DNFB (ear thickness and histopathology), phagocytosis, microbicidal activity, chemotaxis, and T-cell subpopulations (optical density—-ELISA vs. cellular radioactivity—1 µCi of [3H] thymidine after 3 days).	There was a tendency to increase NO production (without statistical significance) by PA at 0.25 µM (3.5 ± 2.3 µM). PA did not restore proliferation in lymphocytes obtained from lymphoma-bearing animals (with increased CD4+ CD25+ T cells in tumor-bearing mice). Higher concentrations inhibited the proliferative response. Positive results were obtained with POH.	[141]
Apoptosis/in vitro	PA (0–5 mM, 24 h) treatment on human non-small-cell lung cancer cells (A549, H520): apoptosis assessed by DNA analysis. At IC_50_ concentrations (3.6 mM, 24 h): Combined treatments with cisplatin (Cis, 1 h) or radiation (1–6 Gy) as sensitizers. Cell viability by Alamar blue. Apoptosis by flow cytometry, proteins by Western blotting and ELISA.	PA did not inhibit proliferation, but viability decreased at 2.5 and 3 mM (IC_50_ 3.6 mM). Pre-treatment (24 h) with PA reduced survival by 30% (A549). Radiation (5 Gy) resulted in 60% survival, dropping to 20% with combined treatment. PA induced a block in the S and G2/M phases in H520 (24 h). Apoptosis with increasing expression of bax, p21, and caspase-3 activity in both cell lines.	[9]
Cell membrane interactions/in silico	Extensive and cumulative molecular dynamics simulations (>2.5 ms) for PA in a zwitterionic lipid bilayer model.	PA causes large-scale membrane thinning, suggesting a lytic mechanism. It is potent in disrupting lipid group packing and modifying the dipole orientation of the main group, bringing putative support to antimicrobial activity.	[142]
Protective action against effects of radiation/in vivo	PA: 50 µmoles/kg bw, i.p. in albino mice exposed to R-γ (6 Gy). Radiation model: (i) white blood cells reduced on the 9th day (1035 cells/mm^3^); (ii) reduction in bone marrow cells on the 11th day (12.5 × 10⁶ cells/femur); (iii) cells positive for α-esterase (674/4000 cells). Elevated levels of pro-inflammatory cytokines IL-1β, TNF-α, CRP; histopathological and DNA analysis (electrophoresis).	PA administration increased bone marrow cellularity (14.8 × 10⁶ cells/femur) and normalized cells positive for α-esterase (941/4000 cells). PA reverted elevated levels of alkaline phosphatase, glutathione-pyruvate transferase, and lipid peroxidation induced by radiation in animal serum and liver. Pa reduced levels of IL-1β, TNF-α, CRP and stimulated CSF and IFN-γ, with increased glutathione in the liver and intestinal mucosa. PA reduced intestinal damage and severe bone marrow damage.	[143]
Immunoregulation/in vitro	CD3+ T lymphocytes, CD3+CD4+ T lymphocytes, and CD3+CD8+ T lymphocytes isolated from the spleen of female C57BL/6J mice (flow cytometry). Treatment with PA (0.5–8 mM): IFN-γ, IL-2, TNF-α, IL-4, and IL-13. T cell proliferation and viability assessed by ELISA.	PA (i) inhibits the production of IFN-γ, IL-2, TNF-α, IL-4, and IL-13 by CD3+CD4+ T cells, and the production of IFN-γ, IL-2, and TNF-α by CD3+CD8+ T cells; (ii) reduces the expression of cell surface markers on CD4+ T cells (CD25 (65%), CD69 (80%), CD40L (65%)) and CD8+ T cells (70%). Viability of CD4+ or CD8+ T cells is not significantly affected (0.5–2 mM). Higher doses induced T lymphocyte death.	[144]
Cell membrane interactions/physicochemical analyses	Calculation of PA partition in membrane model composed of 1,2-dimyristoyl-sn-glycero-3-phosphocholine, which mimics the lipid bilayer of cell membranes and the role they play in biological processes. Techniques employed: differential scanning calorimetry, isothermal titration calorimetry, electron paramagnetic resonance spectroscopy.	PA does not partition into the membrane (unlike other tested perillic derivatives). In general, the membranes are affected in rather subtle ways.	[145]
Cytotoxicity/in vitro	Effect of PA (<0.1% in DMSO) on Na/K-ATPase activity evaluated in U87 and U251 cells (glioblastoma), mouse astrocytes, and VERO cells (non-tumorigenic). Viability: LDH method in the supernatants of treated cells. Apoptosis: flow cytometry. Release of interleukins: ELISA. Positive control: dasatinib.	PA did not affect the selected cells, with a maximum cytotoxicity of 30% at 4 mM.	[146]
Cytotoxicity/in vitro	(S)-POH, (S)-PA, (R)-PA and their sodium salts (S)-NPA, (R)-NPA tested against cancer cell lines (1.0–5.0 mM, DMSO): Caco2, HT-29, HCT-116 (colon carcinomas), MCF7 (breast tumor), K562, Lucena (leukemias), SKMEL (melanoma).	Significant results (IC_50_): (S)-PA: K562 (>10 mM), Caco-2 (2.3 mM), HCT-116: (1.8 mM), HT-29: (2.3 mM). (R)-NPA: Lucena (inconclusive). (S)-NPA: HT-29 (>5.0 mM), HCT-116 (>3.4 mM).	[147]
Antihypertensive, anti-inflammatory/in vitro	Effect of PA on (i) nerve growth factor (NGF); (ii) human bladder cancer cells (HT-1376); (iii) normal human bladder epithelial cells (NHBECs). PA added to the culture medium followed by treatment with IL-1β for 4 h (HT-1376) and 8 h in NHBECs. Total RNA extracted. Cell viability assessed by trypan blue.	PA (and PAL) suppressed the induction of NGF and TNF-α by IL-1β in HT-1376 and normal human bladder epithelial cells.	[148]
Antiviral/in vitro	Effect of PA (50 µM added to a monolayer of infected Vero cells, 24-48-72 h) to assess the replication of HSV-1 (wild-type and mutant). Cytotoxicity: MTT. PCR.	PA (1000–50 µM, 4 doses): EC_50_ 2.84 µM and 1.08 µM for both strains. CC_50_ (wild type) 1812 µM, SI 640. Inhibitory effect continues at 72 h. PA inhibits the release of virions by infected cells, without affecting genomic replication. POH: transformed into PA inside the cells.	[149]
Antitumoral/in silico	PA synthesized from perillaldehyde from *Ammodaucus leucotrichus*: Surflex-docking study against lung cancer: Crystallographic structure of the kinase domain of EGFR protein (associated with cell growth and survival).	PA: low binding affinity with the EGFR protein (score 3.80), below the value for perillyl alcohol (4.18).	[32]
Antidiabetic/in vitro	PA isolated from *Hydrocharis laevigata*: (i) cytotoxicity (MTT) and ROS; (ii) inhibition of α-amylase and α-glucosidase (20–100 µg/mL x acarbose).	PA: Inhibition (x acarbose): α-amylase 22–52%; α-glucosidase: 34–63%.	[150]
Metastasis inhibition and apoptosis/in vitro	Three Pt(IV)/(S)-PA complexes, [(4), (6), (9) on 4 colon cancer cell lines: HCT116, HCT8, RKO, HT29. MTT assay, 72 h after treatment. Reference: oxaliplatin, 5 independent experiments. Calculation of lipophilicity of the complexes and IC_50_ (log k′).	IC_50_ (nM): HCT116: 13.90 ± 1.38 (4)/1.07 ± 0.30 (6)/0.91 ± 0.19 (9); HCT8: 44.41 ± 2.95 (4)/9.52 ± 1.74 (6)/5.03 ± 0.94 (9); RKO: 29.41 ± 4.38 (4)/3.78 ± 0.66 (6)/4.55 ± 1.42 (9); HT29: 29.41 ± 8.85 (4)/4.37 ± 0.32 (6)/8.19 ± 1.75 (9). Pro-apoptotic and pro-necrotic effects. Changes in the cell cycle. Antimigratory activity. Complexes are like or better than PA alone at lower concentrations. Lipophilicity increases the intracellular concentration of the compound.	[151]
Modulation of intestinal ion transport and metabolism; serotonin signaling/in vitro	Incubation with PA (100 μM) using Ussing chamber technique: effect on ionic transport, metabolism, and serotonin signaling in mice ileum. Assessment of (i) tryptophan hydroxylase 1 and monoamine oxidase regulations; (ii) expression rates of serotonin receptors Htr1a, Htr4 and Htr7. Western blot analysis (reference GAPDH).	PA: (i) Tendency to reduce the short-circuit current in the ileum; (ii) positive regulation of tryptophan hydroxylase 1 expression (likewise for *N*-acetylserotonin); (iii) negative regulation of monoamine oxidase A; (iv) positive regulation of the expression of Htr4 and Htr7 genes compared to the control group.	[152]
Antitumoral/in silico	PA tested among terpenoid–peptide conjugates via molecular docking simulations and molecular dynamics with the kinase domain of EGFR and a double mutant. Target receptors implicated in many tumors, specifically lung cancer. Binding affinities determined by Autodock Vina.	More intense apoptosis induced by the peptide conjugates, particularly in cells expressing the double mutant EGFR receptor. PA: binding affinity (kcal/mol): −6.6 (wild type), −5.5 (double mutant). Only three hydrophobic interactions were formed with PA.	[153]

**Abbreviations**. EGFR: Epidermal Growth Factor Receptor. HMG-CoA: 3-Hydroxy-3-methylglutaryl-Coenzyme A. TLC: Thin-Layer Chromatography. Con-A: Concanavalin A. CRP: C-Reactive Protein. DHAP: Dihydroperillic Acid. bw: body weight. 2,4-DNFB: 2,4-Dinitrofluorobenzene. EGFR: Epithelial Growth Factor Receptor. FAME: Fatty Acid Methyl Ester. FBS: Fetal bovine serum. G0, G1, G2: Phases of the cell cycle. GAPDH: glyceraldehyde-3-fosfato dehydrogenase. GC-MS: gas chromatography coupled to mass spectrometry. Gene Htr4: 5-Hydroxytryptamine Receptor 4. Gene Htr7: 5-Hydroxytryptamine Receptor 7. GTP: Guanosine-5′-triphosphate. HSV-1: Herpes Simplex Virus Type 1. HMG-CoA: 3-hidroxi-3-metilglutaril-CoA reductase. HPLC: high-performance liquid chromatography. HPLC-MS: high-performance liquid chromatography coupled to mass spectrometry. i.p.: intraperitonially. Leukotriene B4: One of the leukotrienes produced by leukocytes in response to inflammatory mediators. Fas Ligand: A transmembrane protein expressed on many cell types, including cytotoxic T lymphocytes and natural killer cells, playing a key role in immune regulation and apoptosis induction. LPS: Lipopolysaccharide. M600B: Human Mammary Epithelial Cell Line. mRNA: Messenger RNA (a nucleic acid involved in protein synthesis). MTT: (3-(4,5-Dimethylthiazol-2-yl)-2,5-Diphenyltetrazolium)-Bromide. NGF: nerve growth factor. NIH3T3: NIH/Swiss Mouse Embryo Fibroblast Cell Line. NMR: Nuclear Magnetic Resonance. NO: nitric oxide. p21(ras): Oncogenic Protein Involved in Human Neoplasms (15–20% of all tumors). PA: perillic acid. PAL: perillaldehyde. PCR: Real-Time Polymerase Chain Reaction (for gene expression analysis). PFTase: Farnesyltransferase (enzyme responsible for incorporating farnesol into cellular proteins). PGGTases I and II: Enzymes involved in the incorporation of geranylgeraniol into cellular proteins. PMBC: Peripheral Blood Mononuclear Cell. POH: perillyl alcohol. ROS: Reactive Oxygen Species. SDS-PAGE: Sodium Dodecyl Sulfate Polyacrylamide Gel Electrophoresis. TNF-α: Tumor Necrosis Factor Alpha.

## Data Availability

All data have been presented as an integral part of this manuscript.
